# The perceived benefit of intraoperative stress modifiers for surgeons: an experimental simulation study in volunteers

**DOI:** 10.1186/s13037-021-00294-6

**Published:** 2021-05-29

**Authors:** Sofia Erestam, David Bock, Annette Erichsen Andersson, Eva Haglind, Jennifer Park, Eva Angenete

**Affiliations:** 1 Department of Surgery, Institute of Clinical Sciences, Sahlgrenska Academy at University of Gothenburg, SSORG - Scandinavian Surgical Outcomes Research Group, Sahlgrenska University Hospital, Gothenburg, Sweden; 2 Institute of Health and Care Science, University of Gothenburg, Gothenburg, Sweden; 3 Department of Orthopedics, Sahlgrenska University Hospital, Gothenburg, Sweden; 4 Department of Surgery, Region Västra Götaland, Sahlgrenska University Hospital, Gothenburg, Sweden

**Keywords:** Stress, Surgical simulation, Work break, Saliva cortisol, Patient safety

## Abstract

**Background:**

During surgery, surgeons often work under stressful conditions, which could affect patient safety. Reducing intraoperative stress for surgeons could benefit surgeons and subsequently patients. It is difficult to study stress and stress relief in real life situations due to the multitude of confounding factors. The aim of this study was to evaluate simulated intraoperative stressors on surgeons’ stress levels and the effect of an intervention (pause including a sugar-containing drink) during standardized experiments (simulated operations).

**Methods:**

An experimental interventional study was conducted using a simulator. The healthy surgeon volunteers were randomized to intervention and control in a cross-over design. Primary endpoint was salivary cortisol difference between a pause including a sugar containing drink (intervention) and controls. Secondary endpoints were change in heart rate, change in self-perceived stress measured by the State Trait Anxiety Inventory (STAI), and experience of the intraoperative pause. Endpoints were calculated with a mixed effect analysis of covariance (ANCOVA) model.

**Results:**

Seventeen surgeons performed 32 experiments. There was no statistically significant difference in salivary cortisol between simulations with and without a pause including a sugar-containing drink; percent reduction, 8% (0.92 (95%CI:0.72;1.18)), *p*-value = 0.469. The surgeons’ self-estimation of intervention was positive, but there was no statistically significant difference in heart rate or STAI.

**Conclusions:**

The surgeons’ experience of a pause including a drink was positive but there were no differences in physiological outcomes of the intervention. Lessons learned from this study could contribute to optimizing design of future studies.

**Trial registration:**

Clinicaltrials.gov NCT04626648, Registered November 6, 2020, retrospectively registered.

## Background

In high-risk environments such as the operating room (OR) the ability to recognise and manage stress in oneself and in others is an important non-technical skill. Stress among professionals in the OR has been linked to decreased patient safety as it has been described to affect surgical performance and intraoperative teamwork negatively [1–6]. Knowledge of factors that influence the level of intraoperative stress among members of the operating team are important.

Professions within the OR team have diverse experiences regarding stressors [2, 5, 7–10]. Surgeons may regularly be exposed to various stressors during surgery such as poor teamwork, distractions/interruptions, patient factors, time pressure/management, technical problems, equipment problems, and personal factors [5, 9, 10]. The amount of intraoperative stress and surgeons’ coping strategies could affect surgical performance [11]. Intraoperative stress may also cause fatigue, which may have negative effects on cognitive performance, motor skills, communication and social skills [6, 12–15]. Among surgeons, stress has sometimes been perceived as a sign of weakness and as something that does not affect “me”, but may affect other surgeons [5, 16].

Stress is known to increase levels of cortisol, thus reducing these levels may be a measurement of reduced stress. Efforts to reduce stress in order to enhance performance has been made in various settings. In sports, attempts have been made to add sugar in drink or food resulting in improved performance of the sport, reduced cortisol levels and improved self-reported improved energy and ability to focus [17–19]. In academia similar interventions have been shown to improve scholastic achievement for students [20].

Several aspects of intraoperative stress have earlier been assessed in laparoscopic simulators [6, 21, 22]. Studies have also been conducted on different sorts of intraoperative pauses in various settings and the results indicate that intraoperative pauses reduce stress levels, surgical errors and physical discomfort without prolonging the operating time [11, 23–25]. One group has reported reduction in salivary cortisol and fewer intraoperative events when the surgeons were randomized to 5 min pauses every 30 min compared with no pauses [23]. An intraoperative pause routine together with a sugar-containing drink was introduced and in a retrospective evaluation we found that the surgical team appreciated the intervention. Surgeons reported that they handled problems in a better way and felt refreshed. Most of the surgeons and scrub nurses reported increased patient safety by an intraoperative paus [25].

## Methods

Based on the hypothesis that intraoperative pauses every other hour including a sugar-containing drink would decrease surgeon’s stress levels, the aim of this study was to compare physiologic stress levels measured as cortisol and heart rate, in relation to intraoperative stress and how they was affected by a pause including a sugar-containing drink during simulated operations.

This experimental interventional study with a two-period crossover design was conducted to assess the effects of an intraoperative pause on stress during simulated operations.

### Study participants

**Fig. 1:**
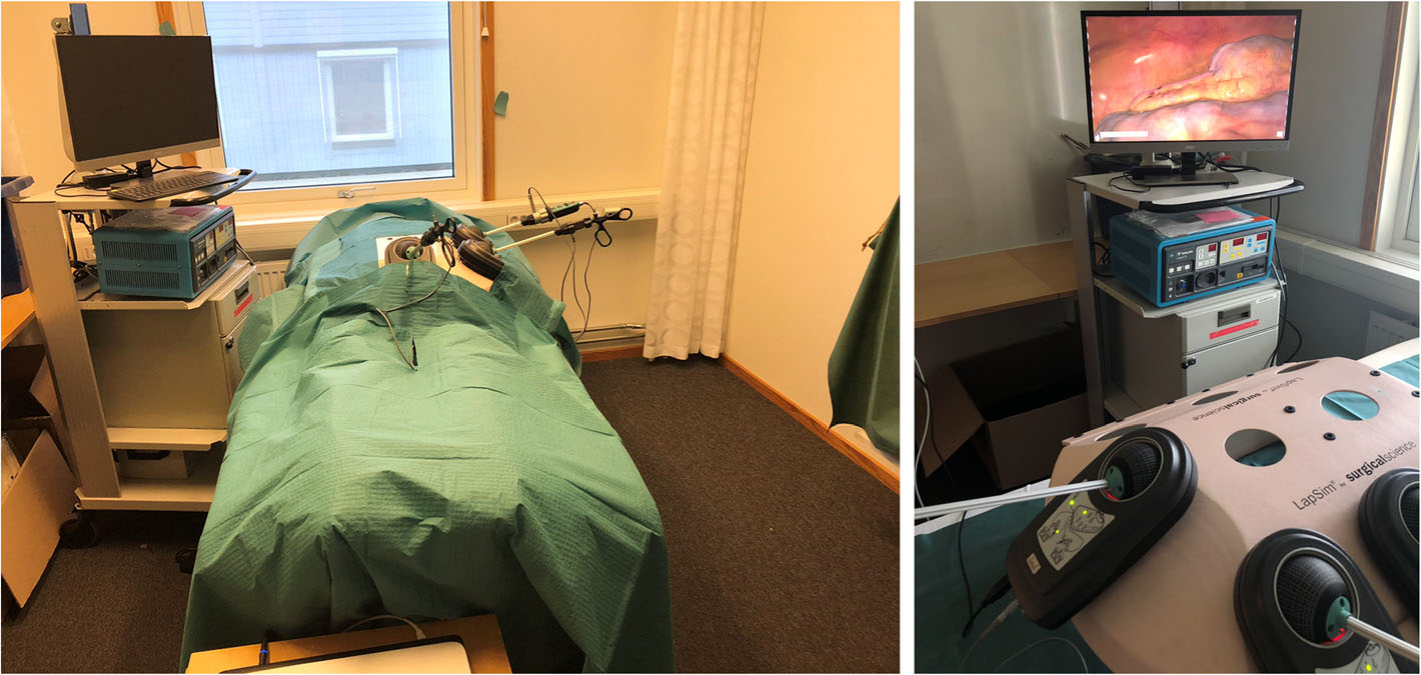
The simulator set-up

Study participants performed the simulated surgery using a two dimensional monitor and instruments similar to clinical surgical laparoscopic equipment (Fig. 1). They were dressed in regular scrubs i.e. scrub cap, surgical gown, gloves and surgical mask, as during regular surgical procedures.

**Fig. 2:**
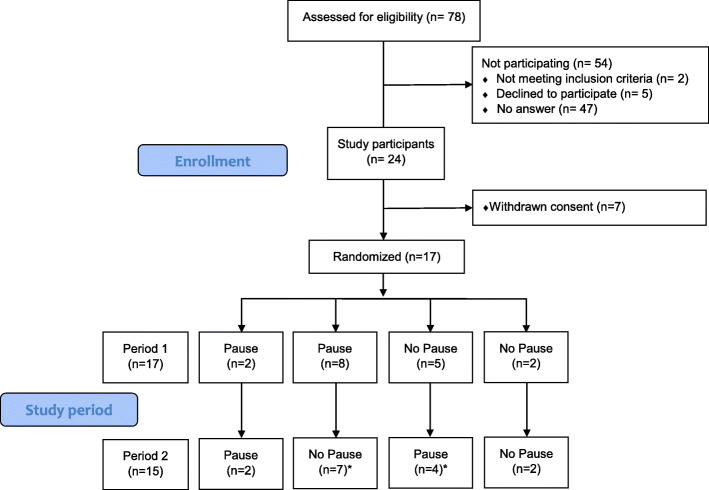
Flow Chart. *Two surgeons did not participate in the second period due to practical reasons

Inclusion criteria for participants were surgical residents or surgeons with maximum 5 years of experience as surgeon (post residency), with employment within Region Västra Götaland. Based on the surgeons’ self-assessment the participants were required to have basic laparoscopic skills and to be able to perform an uncomplicated laparoscopic appendectomy independently. All 78 surgeons who met the inclusion criteria were invited to participate in the study (Fig. 2). One of the authors, SE, enrolled all participants by sending emails with study participant information at two different occasions. Exclusion criteria were: diabetes, Addison’s disease, medication with steroids or medication that affected heart rate (beta-blockers, calcium antagonists, antiarrhythmics, and digitalis). Smokers were also excluded.

### Setting

Simulated operations took place in a specific room at a University Hospital in Sweden. The room was organized in a similar way to an operating room. The surgical procedures were performed in a laparoscopic simulator, LapSim® with the software TeamSim® provided by (Surgical Science, Gothenburg, Sweden, https://surgicalscience.com/) [26]. (Fig. 1).

The included study participants operated in the simulator on two different occasions (periods). Each period consisted of four surgical procedures, performed in the same order at both periods (appendectomy, cholecystectomy, retrocecal appendectomy, and cholecystectomy). Stressors were introduced at approximately the same time points during each period, according to a specific manual. Included stressors were bleeding, fog on the camera lens, “black screen”, and diathermy without function. Stressors were added manually by the attending researcher (SE) using TeamSim® software.

**Fig. 3:**
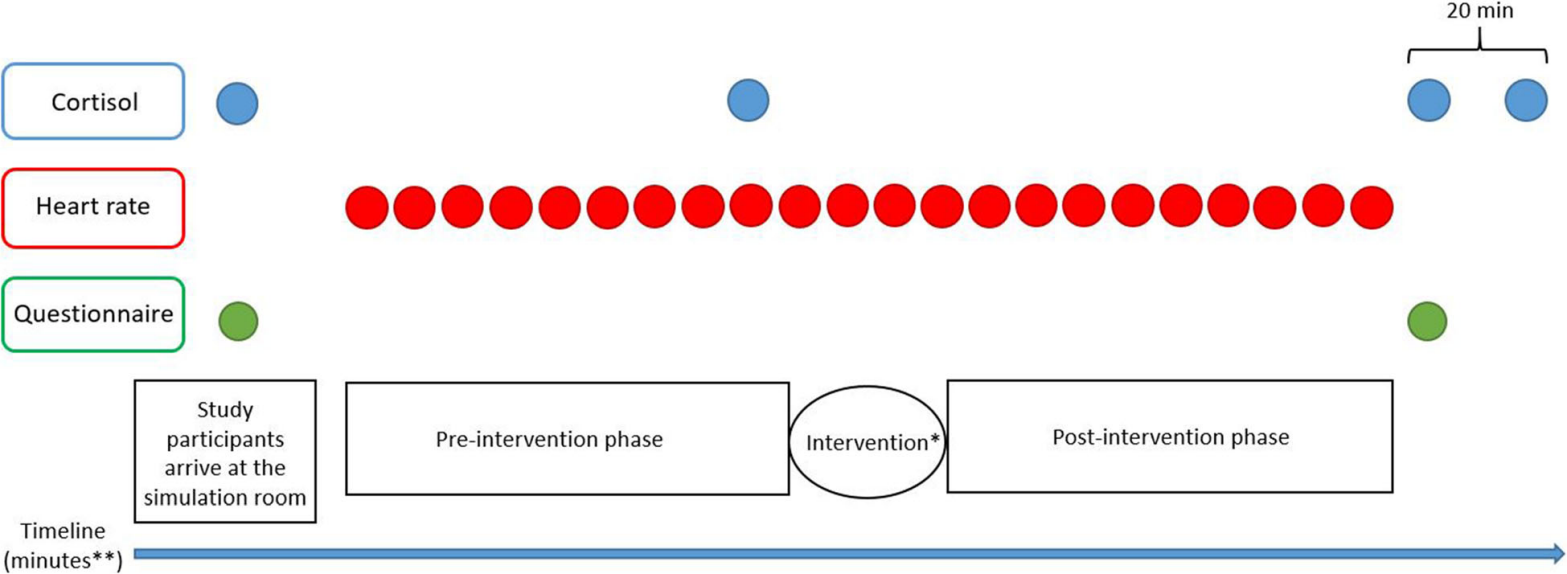
Timeline for measurements. *Intervention = pause including a sugar-containing drink or control = no pause. ** Total time for simulations differed between 0:40 h to 2:02 h

Each period was divided into two different phases, the pre-intervention phase and the post-intervention phase. The periods were performed either without an intraoperative pause (control) or with a three-minute long intraoperative pause, including a sugar-containing drink (intervention). The drink was served with a straw by one of the researchers and the study participants kept their surgical gown and gloves on during the pause. (Fig. 2, Fig. 3).

The sequences analyzed were intervention (with pause) and control (no pause).

Study participants were instructed to not eat, drink (other than water) or use tobacco (snuff) 1 h before each experiment, in order to decrease the risk of affecting their cortisol levels. The diurnal variation in cortisol was taken into account by having the study participants start at the same time point (9 am or at 1 pm) at both simulations.

### Randomization

A computer-generated block-randomization was performed by the statistician (DB). Block-size was unknown to the study participants, who were randomized into one of four sequences (Fig. 2). The majority of the participants were randomized to the sequences: ‘pause - no pause’ or ‘no pause – pause’. Inclusion to the groups ‘pause – pause’ or to ‘no pause - no pause’ was done in order to blind the study participants from knowing if their second simulation would be performed with or without pause (Fig. 2). Allocation was concealed by sequentially numbered, opaque, sealed envelopes. Researchers and study participants did not know their randomization until the time for intervention, when the researcher opened the envelope (Fig. 3).

### Data collection

To measure stress salivary cortisol, heart rate and questionnaires were used. Salivary cortisol was collected with a cotton swab, The Salivette® Cortisol (Sarstedt, Nümbrecht, Germany https://www.sarstedt.com/) [27]. A laboratory at the University Hospital analyzed the saliva samples. Salivary cortisol was collected during each period at four different occasions. The first cortisol was collected before the simulation started. The second cortisol was taken between the pre-intervention phase and the post-intervention phase. The third cortisol was taken immediately after the end of the simulation, and the fourth cortisol was taken 20 min after end of simulation. (Fig. 3).

Heart rate (HR) was measured continuously during simulations (Fig. 3). The assessment was made by a pulse-band worn by each study participant (Polar H10 heart rate sensor), data was extracted from https://flow.polar.com/ [28].

Before and after each period participants filled out questionnaires (Fig. 3). Before the first simulation demographic questions, questions on surgical experience, and previous training in the LapSim® were collected. To assess an individual’s acknowledgement of how their performance was influenced by stressors the factor “stress recognition” in the Safety Attitude Questionnaire (SAQ) was used [29]. To measure self-perceived stress the State Trait Anxiety Inventory (STAI) was used [30]. The short-version containing six questions has been used frequently in stress assessment among surgeons [9, 21, 31–36].

During each period, information on time and type for each added stressor was collected in record forms, as was time for salivary cortisol sampling and start/stop times for the simulated operations.

### Sample size

The primary endpoint was change in salivary cortisol (log-concentration) from baseline to average, based on two pre-intervention and two post-intervention samples (Fig. 3). Assuming a conservative estimate of 0.5 for the correlation, and an intra-individual standard deviation of 0.68 for a single measurement [37]. Then, using the summary statistic approach of Frison and Pocock [38] with 17 evaluable subjects, there would be 80% power to detect a true reduction of 35% [21, 31] in mean cortisol due to an intraoperative break with a two sided test at 5% significance level.

### Statistical analysis

#### Primary endpoint

The primary endpoint was change from the baseline salivary cortisol sample in average log-concentration (nanomole (nmol)/l), based on the two pre-intervention and the two post-intervention cortisol samples (Fig. 3). A secondary outcome was maximum of the two post-intervention measurements.

A mixed effect analysis of covariance (ANCOVA) model with treatment (intervention or control), period and sequence as fixed effects, and subject nested within sequence as a random effect, was used. Baseline mean log cortisol from each period was included as a covariate. Least squares means were calculated for each treatment as well as the difference between an intraoperative pause vs. no pause. The estimates were antilog-transferred back to the original scale to obtain the geometric means and the geometric mean ratios and 95% confidence intervals.

Change from pre-intervention to post intervention phase in heart rate (beats/min), as well as change in STAI score from pre- to post intervention, was analyzed on the log scale with the same statistical model. Questions from earlier studies on stress and surgical performance were used [25, 39]. Subjective measurements by the questionnaire were assessed by Likert scales.

## Results

Out of 78 surgeons (residents or within 5 years of fulfilled residency) seventeen volunteered to participate. They were randomized into one of four different sequences and performed in total 32 simulations. Two of the surgeons did not participate in the second period due to practical reasons (Fig. 2, Table 1). The inclusion was open from June to October 2019 and the study was performed between September and December 2019.

**Table 1:** Demographics and questionnaires

Study participants *n* = 17	Period 1 *n* = 17	Missing	Period 2 *n* = 15	Missing
Age (yrs) median (IQR)	35 (6)			
Sex (n)				
Male	8		6	2
Female	9		9	
Surgical Experience				
Residents	11			
Specialist	6			
BMI median (IQR)	23 (3.1)	1		2
Waist (cm)	80 (13)			2
Previous training in LapSIM ® (n)				
Yes	10			
No	7			
Previous experience of playing videogames (n)				
Never	4			
More seldom than once a month	6			
At least once a month	2			
At least once a week	2			
Every day	3			
Tobacco 12 h (n)				2
Smoked				
Snuff	1		2	
Nicotine patch/ chewing gum				
Alcohol				
Not applicable	16		13	
History of smoking (n)				
I have never smoked	14			
Previous smoker	3			
I smoke				
History of using snuff (snus) (n)				
I have never used snuff	11			
I have stopped using snuff	1			
I use snuff <1x/week	3			
I use snuff	2			
STAI score median (IQR)				
Male	46.67 (18)	2	38.3 (23)	2
Female	36.7 (12)		33.3 (12)	
SAQ Stress recognition score median (IQR)	62.5 (23.44)			
Saltin Grimby, physical exercise (n)				
Sedentary	1			
Some Physical activity	6			
Regular physical activity	9			
Regular hard physical training	1			

Total time for simulations differed between 0:40 h to 2:02 h. There was a time difference between the first median (IQR) 1:39 h (0.33) and second simulation median (IQR) 1:06 h (0.10) (Table 2). Time from awakening to first salivary cortisol differed between 0:28 h to 9:02 h. The intervention (the tree minute pause) took place between the pre-intervention phase and the post-intervention phase, which occurred between 0:22 h and 1:26 h after simulation start. Time from intervention to end of simulation and the third salivary cortisol sample differed between 0:19 h and 1:02 h (Table 2).

**Table 2:** Time measurements

Study participants *n* = 17	Period 1 *n* = 17	Missing	Period 2 *n* = 15	Missing
Time for periods (h) median (IQR)	1.39 (0.33)		1:06 (0.10)	
Time from awakening to first salivary cortisol (h) median (IQR)	3:41:59 (4:30:30)		3:02:00 (4:22:59)	2
Time from pause to cortisol 3 (h) median (IQR)	0:42 (0:14)		0:32 (0:09)	2

**Fig. 4:**
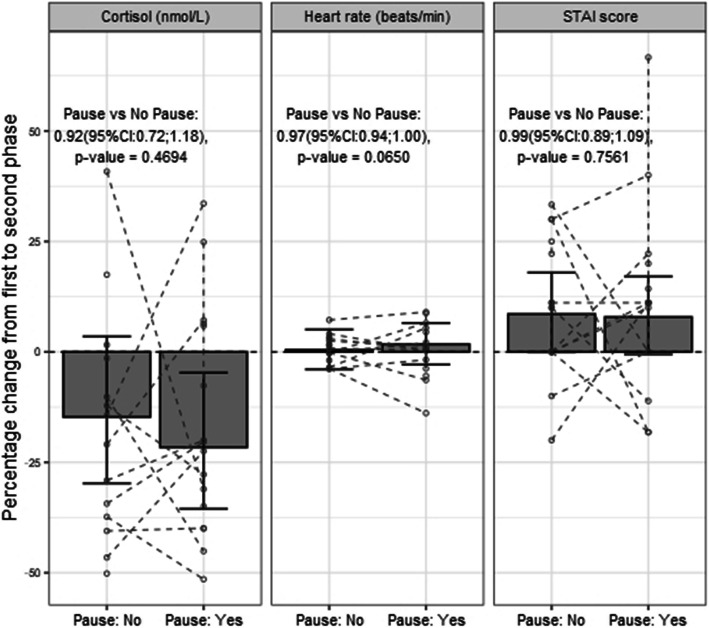
Change in Cortisol, heart rate and STAI score from pre-intervention phase to post-intervention phase

A total of 122 samples of salivary cortisol from 17 participants were analyzed. Results from five of the initial samples were missing due to insufficient amount of saliva, and one sample was lost (Table 3). Salivary cortisol at the pre-intervention phase was median (interquartile range (IQR)) 4.9 (3.5) nmol/l. During the post-intervention phase, after an intraoperative pause, salivary cortisol was median (IQR) 4.6 (2.10) nmol/l and in the control group median (IQR) 4.3 (2.39) nmol/l (Table 3). There was no statistically significant difference in the primary endpoint salivary cortisol between the intervention and the control group, mean ratio 0.92 (95%CI:0.72;1.18) (Table 4, Fig. 4). Analysis of maximum cortisol was calculated but did not differ from mean cortisol.

**Table 3:** Primary and secondary outcome

Median (IQR)	Pause	No Pause	Total
Salivary Cortisol (nmol/l)			
Pre-intervention phase	5.1 (3.40)	4.8 (4.65)	4.9 (3.50)
Post-intervention phase	4.6 (2.10)	4.3 (2.39)	4.5 (2.35)
Heart Rate (beats/min)			
Pre-intervention phase	91.5 (21)	89.5 (30)	93.0 (25)
Post-intervention phase	90.8 (18)	91.8 (29)	91.8 (20)
STAI score			
Pre-intervention phase	33.3 (10.83)	35.0 (10.00)	33.3 (10.00)
Post-intervention phase	41.7 (24.17)	38.3 (26.67)	40 (16.67)

**Table 4:** Percental change from pre-intervention, primary and secondary outcome

	Percent change 95% CI	Yes vs. No	*p*-value
Salivary Cortisol			
Pause: Yes	−21.6%(−35.5 - -4.7)	0.92 (95%CI:0.72;1.18)	0.4694
Pause: No	−14.7%(−29.8–3.5)		
Heart Rate			
Pause: Yes	0.4%(−3.7–3.0)	0.97 (95%CI:0.94;1.00)	0.0650
Pause: No	2.6%(−0.83–6.1)		
STAI			
Pause: Yes	7.9%(−0.56–17.1)	0.99 (95%CI:0.89;1.09)	0.7561
Pause: No	8.6%(0.1–18.0)

The surgeons’ self-perceived assessment of taking an intra-operative pause including a sugar-containing drink was positive. Nine of 16 surgeons experienced that the pause hade made them handle problems in a better way, five did not know, and 14 of 16 surgeons reported feeling more alert after intervention (Table 5).

**Table 5:** Surgeons’ self-perceived assessment of taking a pause

Has the pause made you handle problems in a better way? (n)	yes *n* = 9 no *n* = 2 I don’t know *n* = 5
Do you feel more alert after a pause? (n)	yes *n* = 14 no n = 1 I don’t know *n* = 1

Thirty-one measures of heart rate were analyzed, one measurement was lost due to technical failure. There was no statistically significant differences in heart rate between simulations with intervention compared with controls 0.97 (95%CI:0.94;1.00) (Table 3, Table 4, Fig. 4).

Changes in STAI-score was calculated for thirty simulated operations, two STAI-score was not filled out. STAI-score compared between simulations with interventions and controls did not differ 0.99 (95%CI:0.89;1.09) (Table 3, Table 4, Fig. 4).

## Discussion

In this study, we did not find that an intraoperative pause resulted in a reduction in stress amongst surgeons using physiological measures such as salivary cortisol and, heart rate or a self-assessing questionnaire (STAI). However, a majority of the participating residents and surgeons reported that they felt more alert and that a pause improved their problem-solving skills.

Our hypothesis was that an intraoperative pause would decrease surgeons’ objective and subjective stress levels. Salivary Cortisol, HR and STAI have been validated, where the different assessments correlates and can be used to capture responses to stress during surgery [21, 31]. Although we did not find any association between the intraoperative pause and salivary cortisol, heart rate or STAI, previous studies on stress in combination with intraoperative pauses have reported associations between self-assessed stress, salivary cortisol, heart rate and intraoperative pauses [23, 24]. Engelmann, Schneider [23] and Hallbeck, Lowndes [24] all used several micro breaks as their intervention, whereas the intervention we studied was one intraoperative pause lasting 3 min, including a sugar-containing drink.

The effect of sugar on stress and performance has to our knowledge not been studied in a surgical environment before. In sports, athletes who ingested sugar-containing drinks instead of water during competitions increased their performance in combination with a reduction in cortisol [17]. We expected sugar to have a positive effect on surgeons’ stress levels, but it may have affected our salivary cortisol samples negatively. Cortisol is released into the circulation by activation of the Hypothalamus-Pituitary-Adrenal (HPA) axis and peaks usually 20–30 min after the introduction of stress [40]. The initial study design was a two-hour operation with the pause after 1 h of surgery. The reality was that the simulation time differed between 0:40 h to 2:02 h, as some surgeons were faster than others. This could have had impact on the cortisol as the third cortisol sample was collected 0:19 h to 1:02 h after a pause with a sugar-containing drink.

Time from awakening to first salivary cortisol (Table 1) had a substantial variation (0:28 h – 9:02 h). This could also have affected the results as salivary cortisol normally peaks about 30–45 min after awakening and declines throughout the day [41]. In a supporting analysis cortisol diurnal curve was taken into account by adding simulation time (9 am or 1 pm) as a factor in the statistical model, however this did not change the result.

It is also possible that our design was insufficiently stressful for the study-participants included in this study. The LapSim® simulator is primarily constructed as a teaching tool for residents to shorten their laparoscopic learning curve [42, 43]. Although not all participating surgeons had used the LapSim® previously, it is possible that they had too much surgical experience to perceive the simulations as stressful. In this study, we included residents and surgeons with a maximum of 5 years of surgical experience since we aimed to include study participants who were similar in their laparoscopic experience and thereby reducing inter-individual variability. Including more experienced surgeons would have increased the “possible to include” group but we refrained from this to decrease possible variability in stress responses. More experienced surgeons probably will have developed coping strategies against intraoperative stressful events to a larger extent than less experienced ones even if this is not universal [10, 44].

The sample size calculation showed that we needed to include 17 study participants to have the power to detect a true reduction of 35% in salivary cortisol, based on cortisol decrease in two previous studies [21, 31]. Although we included 17 residents and surgeons, we did not have comparable data for all of them, and our study only demonstrated an 8 % reduction in salivary cortisol. A longer accrual period and more participants would have been preferable. In order to definitely describe a cause-effect relationship between stress, cortisol and anti-stress effect of a pause and drink intervention it would be of interest to perform a study with lessons learned as the subjective measures indicate that a pause is beneficial. The results concerning surgeon experience of a pause and drink are similar to our previous retrospective study, where 75% of the surgeons reported that they handled problems in a better way after a pause, and 93% felt more alert after a pause [25]. Effects experienced by those participating in experiments, studies or treatments should always be respected.

Another reason for future studies on the subject intraoperative stress is the fact that surgeons have reported that stress often is seen as a sign of weakness within the surgical community [5, 16]. At the same time many surgeons indicate that stress does not affect their personal surgical performance [5, 16]. To assess our study participants’ individual understanding of the impact of working in a stressful environment, the SAQ stress recognition domain was included in the baseline questionnaire. Stress recognition has been defined as “the extent to which individuals acknowledge personal vulnerability to stressors such as fatigue, personal problems, and emergency situations” [29, 45].

Strengths of this study include the experimental setting, with standardized “operations”, standardized stressors and a randomized design where each participant was blinded for each experiment (intervention or control). The experiment leader was also blinded up to the moment for the intervention (or not). In connection with this a limitation was that times to complete the standardized operations differed which could have influenced tiredness, possibly a stressor. One limitation was that the sample size was insufficient. Some of the surgeons did not participate in the second period, and some of the cortisol samples could not be analyzed. Another possible weakness in this study was the above-mentioned possibility that the experimental set-up was insufficiently stressful. Also, although the effort was to make the simulations as genuine as possible, there were several disparities from operations in an OR. In real life the surgeon has the entire operating team to rely on, in this study the surgeon was alone and had to solve every problem by him/herself - an added stressor compared to real life. In an effort to simulate real life in an operating room the participants were encouraged to talk to the experimental leader as if she was acting as scrub nurses. One of the main issues during simulations was that the simulator at several occasions stopped working, which led to restarting of the computer. Whether this acted as a stressor for the participants or only for the leader of the experiment is unclear.

## Conclusions

In this study an intraoperative pause including a sugar-containing drink during simulated operations did not significantly change physiological responses to surgeon stress levels. However, the intervention was appreciated by surgeons who felt that their problem-solving abilities were improved. The lessons learned from this study could be of benefit for a future study with even more realistic stressors, one starting time for all and a larger study population.

## Data Availability

Not applicable due to the small sample size hence the possibility for persons familiar with the study to identify the study participants.

## Abbrevations

CRF: Clinical record form
CI: Confidence interval
HR: Heart rate
HPA: Hypothalamus-pituitary-adrenal
IQR: Interquartile range
l: Litre
nmol: Nanomole
OR: Operating room
SAQ: Safety attitude questionnaire
STAI: State trait anxiety inventory
